# The Health and Retirement Study: Analysis of Associations Between Use of the Internet for Health Information and Use of Health Services at Multiple Time Points

**DOI:** 10.2196/jmir.8203

**Published:** 2018-05-25

**Authors:** Hyunju Shim, Jennifer Ailshire, Elizabeth Zelinski, Eileen Crimmins

**Affiliations:** ^1^ USC Davis School of Gerontology University of Southern California Los Angeles, CA United States

**Keywords:** health information technology, health services, disease management, chronic disease, geriatrics

## Abstract

**Background:**

The use of the internet for health information among older people is receiving increasing attention, but how it is associated with chronic health conditions and health service use at concurrent and subsequent time points using nationally representative data is less known.

**Objective:**

This study aimed to determine whether the use of the internet for health information is associated with health service utilization and whether the association is affected by specific health conditions.

**Methods:**

The study used data collected in a technology module from a nationally representative sample of community-dwelling older Americans aged 52 years and above from the 2012 Health and Retirement Study (HRS; N=991). Negative binomial regressions were used to examine the association between use of Web-based health information and the reported health service uses in 2012 and 2014. Analyses included additional covariates adjusting for predisposing, enabling, and need factors. Interactions between the use of the internet for health information and chronic health conditions were also tested.

**Results:**

A total of 48.0% (476/991) of Americans aged 52 years and above reported using Web-based health information. The use of Web-based health information was positively associated with the concurrent reports of doctor visits, but not over 2 years. However, an interaction of using Web-based health information with diabetes showed that users had significantly fewer doctor visits compared with nonusers with diabetes at both times.

**Conclusions:**

The use of the internet for health information was associated with higher health service use at the concurrent time, but not at the subsequent time. The interaction between the use of the internet for health information and diabetes was significant at both time points, which suggests that health-related internet use may be associated with fewer doctor visits for certain chronic health conditions. Results provide some insight into how Web-based health information may provide an alternative health care resource for managing chronic conditions.

## Introduction

### Background

Using the internet for health information has the potential to be a tool for health management [1]. Substantial evidence supports the idea that people improve their knowledge and ability to manage their own health by using Web-based health information [2-4]. Some studies find that more than half of patients using online tools report that they believed health information on the Web was as good as information from their primary physicians [5].

However, empirical tests of the association between the use of the Web-based health information and the use of medical care from a physician among older adults in the United States have been limited to mostly cross-sectional analyses. Previous literature suggests that the use of the internet for health information is associated with more visits to a doctor among older adults in the United States [6,7]. Research has also shown that health-related internet users are younger, more highly educated, have better cognitive functioning, better health literacy, and are sicker [7,8-11]. However, because most of these studies among older persons have been largely descriptive with a cross-sectional design, whether the effect of using the internet for health information on health service use is independent or correlated with other concurrent factors in the model remains unclear. In addition, the interactions between health-related internet use and different disease conditions on health care utilization have not been tested despite its implications for self-care management.

Due to these limitations, the evidence on the effect of using the internet for health information remains mixed. Some studies have reported that searching for health information on the Web may offer older adults an opportunity to engage in more patient-centered interactions with physicians and promote self-health management [12], such as through the use of online discussion boards or searching for information on diseases and conditions. Other researchers, however, have found that the use of the internet for health purposes may not be influential on individuals’ offline interactions with their physicians and treatment decision-making [13], and have raised concerns that such activity may exacerbate health-related anxiety among some individuals [14].

### Theoretical Framework

**Figure 1:**
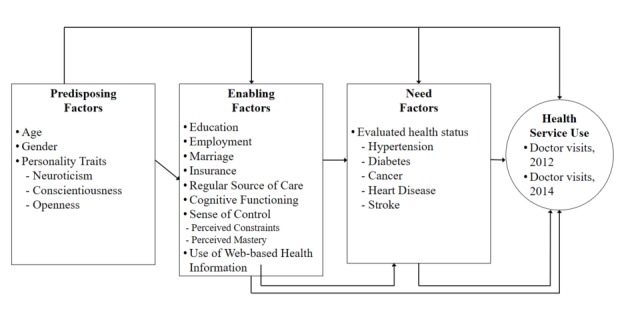
Conceptual framework.

Andersen’s Behavioral Model of Health Services Use is one of the most widely used models that identifies constructs related to the use of health care services [15-20]. This model has been refined and expanded over the years and remains the most highly adopted approach to modeling health service usage [21,22]. The model posits that health care use is influenced by individuals’ predisposing factors, enabling factors, and need factors. Our model is derived from the Andersen model of general health service usage [16,21,22], as described in Figure 1. *Predisposing factors* describe factors that incline people to use health care services (eg, age, gender), which include personality traits in this study. Recent studies have suggested that psychological characteristics are important to include among predisposing factors [23]. *Enabling factors* facilitate health service use (eg, health insurance, regular source of care, use of Web-based health information). For instance, the availability of health-related information has recently emerged as an important enabling characteristic among members of ethnic minority groups [24]. However, applying this model to older people and including the use of health-related information on the Web specifically as an independent factor remains unexplored. Therefore, we include the use of Web-based health information as one of the enabling factors that predispose people to health service use. Sense of control is also included as a psychosocial characteristic that may be enabling, as sense of control has been shown to be a key factor influencing both optimism and discomfort in individuals’ readiness to use technology [25], and those with high sense of control were found to be more likely to seek Web-based health information [26-28]. *Need factors* include both self-perceived and evaluated health needs (eg, comorbid conditions, self-reported health). This study examines reports of 5 doctor-diagnosed health conditions (ie, hypertension, diabetes, cancer, heart disease, and stroke) as an indicator of need.

### Objective

The primary purpose of this study was to examine whether the health-related internet use is linked to health service usage at 2 time points in a nationally representative survey. Examining the use at multiple time points helps to clarify whether using the internet for health information is associated with subsequent use in health care utilization or whether the association is only concurrent. Additionally, we also explore whether having a chronic health condition and the use of Web-based health information are synergistic by testing their interactions, which have not been examined in prior studies. Examination of the synergistic effect of different diseases and the use of the internet for health information on physician visits may have important implications for developing future tools for self-care management. The findings of this study provide the first evidence on the association between the use of the internet for health information and health service use at multiple time points and add to our understanding on how different diseases are related to this association. Using nationally representative data from the Health and Retirement Study (HRS), we aim to (1) test whether the use of Web-based health information and health service use are associated at concurrent and subsequent times (2) and examine whether the association is different among individuals with different chronic conditions.

## Methods

### Data

We used data from the HRS, a nationally representative sample of more than 20,000 older Americans aged 50 years and above. In 2012, the HRS included a technology module including a question on the use of Web-based health information that was administered to a random subsample of 1740 participants from the core participants.

Health service use is indicated by responses to a question on the number of visits to a doctor in the past 2 years. We assessed the effect of Web-based health information use on health care utilization both in the period (2012) concurrent with the reported internet use and in the period (2014) after the use, to see whether associations are present subsequent to the reported use of Web-based health information.

We also used psychosocial measures collected in a self-administered leave-behind questionnaire (SAQ) that is also administered to a random subsample of core participants on a biennial basis. The first random half-sample of households received the SAQ in 2010 and the second half in 2012. The combined 2010 and 2012 sample had an overall response rate of 71% [29].

Therefore, we limited the sample to 1737 respondents who responded to the question on the use of Web-based health information. For 2012, 73 cases were missing on visits to a doctor; 265 participants who did not have complete information in 2014 were also excluded. We also removed 251 respondents who did not meet age eligibility (under age 52 years), 34 participants who were missing on covariates, and 123 participants who revealed missing data on 5 psychosocial measures in the SAQ. The final analytic sample comprised 991 persons aged 52 years and above in 2012 who were followed up and had complete information in 2014.

### Measures

#### Health Service Use

Health service use is measured by the number of times participants saw or talked to a medical doctor about their health in the last 2 years, reported in 2012 and 2014. Respondents were asked, “Aside from any hospital stays or outpatient surgery, how many times have you seen or talked to a medical doctor about your health, including emergency room or clinic visits in the last two years?” This question has regularly been used in the literature based on the HRS to assess health care utilization [30].

We categorized the independent variables affecting health service use according to conceptual framework in Figure 1, as predisposing, enabling, and need factors.

### Predisposing Factors

#### Demographic Variables

Demographic variables included age and gender (male or female). Personality traits include neuroticism, conscientiousness, and openness because these 3 personality traits were found to be significant predictors of health-related internet use and health service use in prior studies [7,31]. These traits were derived from participants’ rating of how well a list of words described them (1=a lot, 2=some, 3=a little, and 4=not at all). Response options were reverse coded to indicate higher agreement with each personality construct. Neuroticism was measured by moody, worrying, nervous, and calm (reversed; alpha=.71); conscientiousness with organized, responsible, hardworking, self-disciplined, careless (reversed), reckless (reversed), impulsive (reversed), cautious, thorough, and thrifty (alpha=.71); and openness to experience with creative, imaginative, intelligent, curious, broad-minded, sophisticated, and adventurous (alpha=.78). The composite scores for each trait ranged from 1 to 4, with higher scores indicating more of a given personality trait (see Multimedia Appendix 1). The scores of all items were then averaged within subdimensions of each personality trait. These personality items have been validated for comparability of each adjective item and tested for measurement invariance for assessing personality across different age groups [32,33].

### Enabling Factors

#### Use of Web-Based Health Information

For assessing the use of Web-based health information, respondents were asked, “Do you use websites for finding medical and health information?” in the 2012 Technology Module. The response was binary (1=yes, 0=no) and coded as a dichotomous variable.

#### Sense of Control

We included sense of control indicated by perceived constraints and mastery (see Multimedia Appendix 2). Perceived constraints indicate one’s assessment of external problems or barriers and were measured by participants’ reports of agreement or disagreement with the 5 statements on how they perceive barriers or obstacles in life. Perceived mastery indicates one’s assessment of sense of competence over life circumstances and was measured by agreement or disagreement with the 5 statements. The answers for both questions used a 6-level Likert scale and the composite scores ranged from 1 (strongly disagree) to 6 (strongly agree), with higher values indicating greater agreement. The Cronbach alpha reliability coefficients were high for both constraints (alpha=.86) and mastery (alpha=.89). The scores of all items were then averaged within each of the 2 scales. All the psychosocial measures were coded following the instructions from the HRS psychosocial questionnaire guidelines [34].

#### Other Covariates

We included sociodemographic and other characteristics that facilitate health service uses. Educational attainment is measured with years of completed education, ranging from 0 to 17. Marital status (1=married, 0=not married) and employment (1=working, 0=not working) are dichotomized. Insurance was also a binary variable indicating whether the respondent reported having any type of public or private insurance (0=none, 1=has insurance). Having a regular source of care was assessed with the question that asked “Is there a place that you usually go to when you are sick or need advice about your health?” with a dichotomous answer (1=yes, 0=no). Cognitive functioning is measured by a summary score based on a series of tests including immediate and delayed recall tests (0-20), backward counting (0-2), and a serial 7s subtraction (0-5), with a score ranging from 0 to 27 [35]. This score assessing working memory and attention has been used in in many studies as a composite score indicating cognitive functioning [36,37].

### Need Factors

#### Health Covariates

Health measures include self-reports of whether a doctor ever told the respondent that he or she had a set of chronic conditions: hypertension, diabetes, cancer, heart disease, or stroke. Responses were coded into binary variables indicating the presence or absence of each condition in 2012 and 2014.

### Statistical Analyses

We first examined bivariate associations using *χ*^2^ tests and *F* ratios to examine differences between users and nonusers of Web-based health information on predisposing, enabling, and need characteristics. We then turned to testing the predictive value of the use of Web-based health information for the number of visits to a doctor for both 2012 and 2014. We first estimated the effect of using Web-based health information on doctor visits controlling for predisposing, enabling, and need factors using negative binomial regressions. The examination of doctor visits data showed that the variances were higher than the means within each level of the use of Web-based health information, suggesting overdispersion of count data. Therefore, negative binomial regression models were used to address skewed distribution and overdispersion of the count data [38]. The likelihood ratio test also showed that the negative binomial model fitted better than a Poisson model. This method has been used in prior HRS studies that examined doctor visits as an outcome of interest [30,39]. In addition, interaction terms for the use of Web-based health information and 5 doctor-diagnosed health conditions were included to examine whether the use differentially affects these health conditions to have synergistic effects on doctor visits.

Finally, to test the validity of the overall results and detect any potential type I error of interaction terms, we performed the False Discovery Rate (FDR)-controlling procedure tests. The FDR-controlling procedure test detects the expected proportion of false positives through dividing the number of true null hypotheses rejected by the total number of hypotheses rejected, which can also be regarded as the probability of making even one error if all null hypotheses are true [40]. We applied the Benjamini-Yekutieli method to test whether the significant results were spurious or not, using Stata’s *multproc* command [41]. Stata 13 (StataCorp LP, College Station, TX) is used to estimate all models. Analytic results were weighted using Stata’s survey prefix commands (SVY) to account for complex survey design in terms of differential sampling probabilities, nonresponse, population stratification, and sample weights.

## Results

### Sample Characteristics

Table 1 describes the sociodemographic, health, health care utilization characteristics of the internet users for health information compared with nonusers. Women were more likely to be users of Web-based health information (58.3% of users). The users were also significantly more likely to be younger, married, and in the labor force. Users were more conscientious (3.32 [SD 0.38] vs 3.22 [SD 0.43], *P*=.001) and open to experience (3.04 [SD 0.49] vs 2.87 [SD 0.51], *P*<.001). Users also had better cognitive functioning (17.25 [SD 3.48] vs 15.12 [SD 4.24], *P*<.001), but differences between users and nonusers on disease-related characteristics were mixed. The presence of hypertension and heart disease differed significantly between users and nonusers: 17.8% of users compared with 25.3% of nonusers on heart disease. However, there was no significant difference in the prevalence of diabetes, cancer, and stroke between users and nonusers (Table 1).

### The 2-Time Point Analysis and the Interaction of the Use of Web-Based Health Information

Table 2 presents the results from the negative binomial regression models predicting the number of visits to a doctor in 2012 and 2014. Model 1 and Model 3 present the adjusted models with all predisposing, enabling, and need factors. Among predisposing factors, being female was associated with higher visits to doctors in both 2012 and 2014.

**Figure 2:**
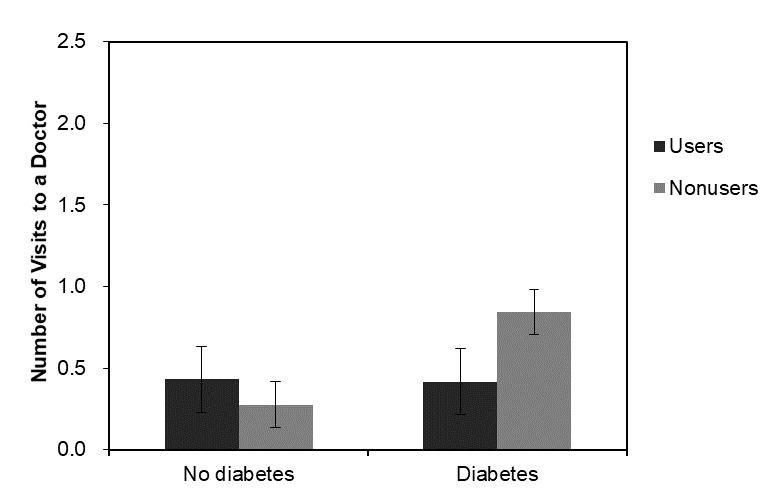
Predicted mean number of visits to doctors and the interaction of the use of Web-based health information and diabetes in 2012.

**Figure 3:**
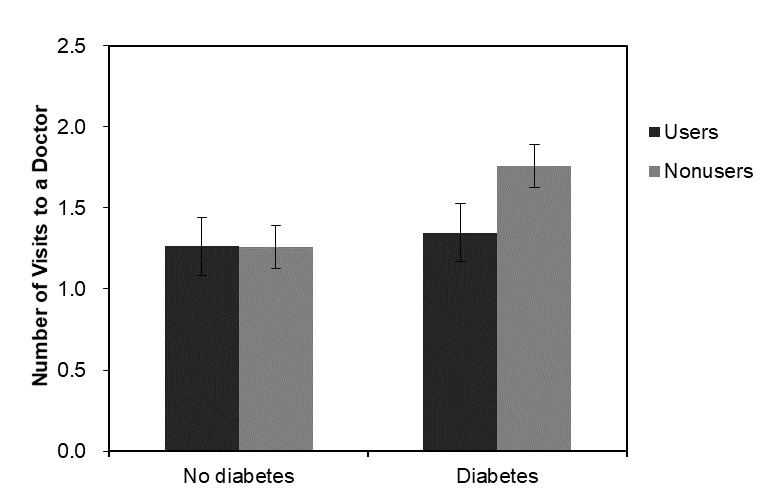
Predicted mean number of visits to doctors and the interaction of the use of Web-based health information and diabetes in 2014.

The use of Web-based health information was associated with significantly more doctor visits in 2012 (*b*=0.189, SE=0.095, *P*=.048), but the association was not significant in 2014 (*b*=0.047, SE=0.091, *P*=.60), indicating that the number of visits to a doctor in the last 2 years reported concurrently is higher among those who report the use of Web-based health information, but the effect on use in the next 2 years is not significant. Model 2 and Model 4 add interaction terms with different disease conditions and the use of Web-based health information. The inclusion of interaction terms in Model 2 and Model 4 showed the synergistic effect of the use of Web-based health information and diabetes at both time points. Having diabetes (*b*=0.561, SE=0.140, *P*<.001) was associated with a higher number of visits to a doctor, but when the respondents used Web-based health information, the predicted number of visits was lower (*b*=−0.575, SE=0.204, *P*=.005). The results were similar in the 2014 models for the main effect (*b*=0.493, SE=0.130, *P*<.001) and the interaction term (*b*=−0.409, SE=0.179, *P*=.022). The interaction between the use of Web-based health information and diabetes on doctor visits is plotted in Figures 2 and 3.

Among predisposing factors, being female was associated with higher visits to doctors across all models. Conscientiousness was significantly associated with fewer visits to doctors in 2012 (*b*= −0.255, SE=0.127, *P*=.04), but not in 2014. Similarly, openness to experience was significantly associated with more doctor visits in 2012 (*b*=0.290, SE=0.100, *P*=.004) only. Among enabling characteristics, being able to work was associated with less number of doctor visits across all models.

To test the validity and robustness of these findings, all estimates were tested for multiplicity using the Benjamini-Yekutieli method with the corrected overall critical *P* value of .002. For the significant results in 2012, having the usual source of care and the main effect of diabetes were rejected with the overall corrected *P* values. For 2014, the main effect of having diabetes and constant were rejected. However, the interaction effect for diabetes remained significant.

**Table 1:** Sample characteristics: Health and Retirement Study (2012). Weighted percentages and weighted means with standard deviation in parentheses.

Variables		Total (N=991)	Users (N=476)	Nonusers (N=515)	*P* value^a^
Female, n (%)		557 (52.9)	290 (58.3)	267 (46.3)	.003
Married, n (%)		603 (64.0)	315 (68.1)	288 (59.0)	.021
Working, n (%)		333 (39.8)	197 (46.3)	136 (31.7)	<.001
**Doctor-diagnosed health conditions, n (%)**					
	Hypertension	613 (55.5)	277 (49.7)	336 (62.7)	.001
	Diabetes	213 (18.6)	98 (17.1)	115 (20.4)	.26
	Cancer	145 (14.8)	77 (15.9)	68 (13.6)	.41
	Heart disease	230 (21.1)	95 (17.8)	135 (25.3)	.019
	Stroke	62 (6.0)	26 (4.5)	36 (7.7)	.10
Any insurance, n (%)		822 (80.6)	388 (78.3)	434 (83.4)	.12
Usual source care, n (%)		862 (87.6)	420 (88.0)	442 (87.2)	.78
Age, mean (SD)		65.31 (9.47)	62.79 (7.78)	68.37 (10.40)	<.001
Years of education, mean (SD)		13.64 (2.58)	14.25 (2.35)	12.90 (2.66)	<.001
Cognitive functioning^b^, mean (SD)		16.29 (3.98)	17.25 (3.48)	15.12 (4.24)	<.001
**Psychosocial factors^c^, mean (SD)**					
	Neuroticism (1-4)	2.02 (0.60)	2.04 (0.62)	2.00 (0.59)	.51
	Conscientiousness (1-4)	3.27 (0.41)	3.32 (0.38)	3.22 (0.43)	.001
	Openness (1-4)	2.96 (0.50)	3.04 (0.49)	2.87 (0.51)	<.001
	Perceived constraint (1-6)	2.00 (1.04)	1.93 (1.09)	2.07 (0.97)	.09
	Perceived mastery (1-6)	4.83 (1.06)	4.88 (1.07)	4.78 (1.04)	.21
**Number of visits to a doctor, mean (SD)**					
	2012	8.53 (13.49)	9.40 (15.76)	7.46 (10.02)	.04
	2014	8.06 (10.15)	8.35 (10.08)	7.71 (10.23)	.41

^a^*P* value indicates significant difference between users and nonusers.

^b^Cognitive functioning indicates average summary score based on a series of tests including immediate and delayed recall tests (0-20), backward counting (0-2), and a serial 7s subtraction (0-5), with a score ranging from 0 to 27.

^c^Psychosocial factors indicate average scores on a scale.

**Table 2:** Negative binomial regression analysis of association between the use of Web-based health information and visits to a doctor (N=991).

Predictor			2012						2014					
			Model 1			Model 2			Model 3			Model 4		
			β	*P*	SE	β	*P*	SE	β	*P*	SE	β	*P*	SE
**Predisposing factors**														
	Age		0.000	.96	(0.007)	0.003	.60	(0.006)	−0.005	.35	(0.005)	−0.005	.34	(0.005)
	Female		0.284	.008	(0.107)	0.289	.004	(0.101)	0.213	.01	(0.085)	0.228	.008	(0.085)
**Personality traits**														
	Neuroticism		0.080	.40	(0.096)	0.084	.35	(0.092)	0.120	.11	(0.077)	0.115	.12	(0.076)
	Conscientiousness		−0.255	.04	(0.127)	−0.254	.03	(0.118)	−0.211	.10	(0.130)	−0.202	.12	(0.130)
	Openness		0.290	.004	(0.100)	0.286	.004	(0.098)	0.060	.56	(0.103)	0.063	.53	(0.102)
**Enabling factors**														
	Use Web-based health information		0.189	.048	(0.095)	0.157	.30	(0.152)	0.047	.60	(0.091)	0.008	.96	(0.157)
	Education		0.018	.34	(0.019)	0.023	.20	(0.018)	0.046	.007	(0.017)	0.046	.006	(0.016)
	Working		−0.265	.038	(0.127)	−0.232	.04	(0.115)	−0.270	.004	(0.092)	−0.271	.004	(0.092)
	Married		−0.094	.39	(0.111)	−0.091	.37	(0.102)	0.010	.90	(0.087)	0.018	.83	(0.085)
	Insurance		0.212	.12	(0.137)	0.192	.14	(0.131)	0.302	.018	(0.127)	0.314	.01	(0.122)
	Regular source of care		0.514	.001	(0.158)	0.518	<.001	(0.143)	0.195	.18	(0.148)	0.196	.18	(0.148)
	Cognition		0.017	.27	(0.015)	0.017	.21	(0.014)	0.008	.56	(0.013)	0.006	.66	(0.014)
**Sense of control**														
	Perceived constraint		−0.049	.36	(0.054)	−0.052	.31	(0.052)	−0.076	.09	(0.046)	−0.079	.09	(0.047)
	Perceived mastery		−0.028	.53	(0.045)	−0.027	.52	(0.042)	0.006	.89	(0.045)	0.003	.94	(0.044)
**Need factors**														
	**Hypertension**		0.254	.01	(0.099)	0.272	.028	(0.123)	0.301	<.001	(0.084)	0.183	.10	(0.113)
		× Use Health Information				−0.042	.81	(0.179)				0.220	.19	(0.168)
	**Diabetes**		0.276	.007	(0.102)	0.561	<.001	(0.140)	0.298	.003	(0.100)	0.493	<.001	(0.130)
		× Use Health Information				−0.575	.005	(0.204)				−0.409	.022	(0.179)
	**Cancer**		0.304	.04	(0.146)	0.130	.42	(0.162)	0.057	.58	(0.103)	0.165	.31	(0.163)
		× Use Health Information				0.256	.33	(0.267)				−0.177	.37	(0.199)
	**Heart Disease**		0.394	.005	(0.139)	0.076	.56	(0.129)	0.212	.02	(0.093)	0.164	.21	(0.132)
		× Use Health Information				0.625	.009	(0.238)				0.120	.48	(0.173)
	**Stroke**		0.197	.40	(0.236)	0.191	.31	(0.188)	0.371	.12	(0.240)	0.396	.24	(0.338)
		× Use Health Information				−0.050	.91	(0.491)				−0.181	.67	(0.430)

## Discussion

### Principal Findings

Almost half (48.0%) of the adults in this national sample of the US population aged 52 years and above used the internet for health information. This estimate is comparable to the national estimates of Web-based health information users reported in other studies, which range from 48.2% [42] to 58.0% [43] for older Americans. To our knowledge, this is the first study to examine the effect of the use of the internet for health information on the health service use among older adults at multiple time points and test the interaction effects with different diseases.

Among the predisposing factors, being female was consistently associated with a higher number of physician visits. This result is consistent with prior evidences on gender differences in the use of the internet for health information and health service use [6,7]. Prior studies found that women had higher health literacy than men, even when adjusting for sociodemographic and health factors [44,45]. Further studies will need to examine more detailed understanding of what motivates women to use health service more. Although the results on conscientiousness are counterintuitive, it is important to note that the significant association between conscientiousness and doctor visits was reported only among older men and not older women [46]. The relationship between personality and health service use remains unclear. There are more studies on diseases and personality, which found conscientious people are significantly less likely to have onset of diseases [47]. Because association between conscientiousness and doctor visits per se has received relatively little attention, further studies may focus on this relationship to clarify this association.

Regarding enabling factors, the use of Web-based health information was associated with more doctor visits at the same period, which is consistent with prior findings [6,7]. However, the association was not significant 2 years later. As most studies focused on examinations of using Web-based health information and doctor visits at one time point, using multiple time points in our study helps to understand how persistent the finding is over time. The lack of a relationship in 2014 indicates that the association is strongest when the use of the internet for health care information and health service use is coterminous, raising the possibility that the initial association was due to either reverse causation or causation by a third factor, such as need.

On the other hand, with regard to need factors, the interaction between the use of Web-based health information and chronic conditions on doctor visits was consistent for all models at both time points. Although the main effect of having diabetes was associated with more doctor visits, the association was negative when the use of the internet for health information was interacted with diabetes, suggesting a reduced number of visits to a doctor associated with being a user of Web-based health information with diabetic conditions. This result remained robust through the multiplicity tests and adds a novel finding to the existing literature.

### Implications and Future Directions

Our study indicates that there may be an opportunity to further promote the development of resources for self-care and health management. Diabetes is a chronic condition that requires self-care as an essential component of management [48]. Due to this, there are many mobile phone apps and devices targeting diabetic patients [49-51], which may offer sources of health management that substitute visits to doctors. For practical reasons, some diabetic patients may prefer using Web-based health information to going to physicians.

Using the internet for health information may enable people with fewer economic resources to manage their conditions in a cost-efficient way. The national evaluation of health care costs for managing diabetes in the United States in 2012 stood at US $245 billion, out of which 9.0% was associated with physician office visits [52]. Therefore, the use of Web-based health information might be used to offset some of the burden. Additionally, online support could provide an opportunity for people with physical disabilities, who otherwise may find it difficult to visit physicians on a regular basis, to utilize health care resources. A recent meta-analysis estimating the risk of physical disability associated with diabetes reported that diabetes nearly doubles the risks of mobility disability [53]. Being able to use Web-based health information may enable older adults to manage their conditions despite physical disability. Finally, using Web-based health information could also be an enabling resource for older people who face linguistic barriers in utilizing health care services. One study found that the health-related internet use enabled Korean immigrant women in the United States to assist with self-care management, ranging from diagnosis and treatment to the recommendation of hospitals by allowing communications free of concerns about language barriers, indicating the potential empowerment by the use of the internet for health information among immigrant populations [54]. As the number of people who cannot speak fluent English is high among older immigrants, this finding has important implications for the use of Web-based health information in relation to health service use.

However, evaluating the effectiveness of the use of Web-based health information for chronic disease management remains to be addressed in future studies. One prior study reported that users of Web-based health information checked their blood glucose more regularly and practiced health behaviors better than nonusers among 344 patients with type 2 diabetes [55]. Yet, another qualitative study of 46 diabetic patients found that most participants passively received information, and although some diabetic patients collected information from Web sources, they sought medical professionals’ confirmation on the reliability and applicability of information [56]. Therefore, it is not clear whether and how using Web-based health information is an alternative or a supplement to conventional health care resources.

There is also a concern regarding the quality of Web-based health information. Some prior studies reported that community-dwelling older adults aged 50 years and above showed general concerns regarding the credibility of Web-based health information and how time-consuming the Web-based health information search can be [57]. These concerns were echoed by a study conducted on caregivers and their older care recipients, which raised questions on the reliability and effectiveness of Web-based health information [58]. Future studies should attempt to address concerns related to the reliability of Web-based health information and its implications for self-care management of chronic conditions for both older adults and their family caregivers. Additionally, the potential for the use of Web-based health information to enable socioeconomically and physically marginalized population to utilize the health care system merits further research attention.

### Limitations

This study has several limitations. First, the questions on doctor visits at the first time point did not indicate a clear timing of the doctor visits relative to the use of Web-based health information. Although we examined doctor visits in both 2012 and 2014 to partly address this issue, this does not completely eliminate concerns in interpreting the findings. There is also a possibility that people may not have accurately responded to the question on visits to the doctor due to memorability reasons with regard to a 24-month time horizon. Second, the relatively simple question on the use of Web-based health information in the HRS also limited the scope of analysis. Detailed information on the purpose for seeking Web-based health information (eg, communicating with physicians, searching for information, taking care of their spouses) would have advanced our understanding on why some older adults seek health information on the internet and why women use Web-based health information more. Despite these limitations in the questions, however, this study has a unique contribution to expand our understanding on the association between the use of the internet for health information and health service utilization by using a nationally representative sample. Finally, the study relies on self-reports of doctor-diagnosed medical conditions which may have some reporting errors. However, previous research has shown relatively high agreement between self-reports of health conditions and medical record reports [59,60].

### Conclusions

This study provides the first evidence on how the use of the internet for health information and chronic health conditions are synergistically associated with doctor visits at 2 different time points. The findings indicate that, although the use of Web-based health information is independently associated with doctor visits concurrently, this effect does not remain significant 2 years later. However, for those with a certain chronic health conditions, using Web-based health information is significantly associated with fewer doctor visits at both time points, suggesting a potential avenue for future research in exploring the implications of the use of Web-based health information among older people. Examination of predisposing, enabling, and need factors, as well as their interactions, adds to our understanding of the relative importance of association between the use of Web-based health information and health service use.

Using the internet for health information may help older adults with a barrier to access conventional health care system—including those with lower socioeconomic resources, physical limitations, and linguistic barriers—to manage their conditions. However, caution needs to be exercised in extrapolating these study findings. Although accessing the health information on the Web may provide an alternative source of health care services, further research will need to evaluate quality and reliability of Web-based health information to elucidate the potential role of using this technology for health management among older people.

## Abbreviations

FDR: False Discovery Rate
HRS: Health and Retirement Study
SAQ: self-administered leave-behind questionnaire

## Appendix

Multimedia Appendix 1 Questions on personality traits (Health and Retirement Study, 2012).

Multimedia Appendix 2 Questions on sense of control (Health and Retirement Study, 2012).
