# Supplementary material for: The Health and Retirement Study: Analysis of Associations Between Use of the Internet for Health Information and Use of Health Services at Multiple Time Points
Source: J Med Internet Res. 2018 May 25;20(5):e200. doi: 10.2196/jmir.8203 (PMC5993973; doi:10.2196/jmir.8203)
Supplement: Multimedia Appendix 1 [file jmir_v20i5e200_app1.pdf]

Appendix 1. Questions on Personality Traits (Health and Retirement Study, 2012)

Q. *Please indicate how well each of the following describes you.*

Response categories: 1=A lot, 2=Some, 3=A little, 4=Not at all

Neuroticism

Q33d Moody

Q33h Worrying

Q33l Nervous

Q33q Calm

Conscientiousness

Q33c Reckless

Q33e Organized

Q33i Responsible

Q33n Hardworking

Q33r Self-disciplined

Q33v Careless

Q33x Impulsive

Q33z Cautious

Q33z_5 Thorough

Q33z_6 Thrifty

Openness to Experience

Q33m Creative

Q33o Imaginative

Q33s Intelligent

Q33t Curious

Q33w Broad-minded

Q33z_3 Sophisticated

Q33z_4 Adventurous

Reverse-coded all items EXCEPT Q33c, Q33q, Q33v, Q33x, and averaged the scores for items within sub-dimensions for each personality trait.
