# Supplementary material for: The Health and Retirement Study: Analysis of Associations Between Use of the Internet for Health Information and Use of Health Services at Multiple Time Points
Source: J Med Internet Res. 2018 May 25;20(5):e200. doi: 10.2196/jmir.8203 (PMC5993973; doi:10.2196/jmir.8203)
Supplement: Multimedia Appendix 2 [file jmir_v20i5e200_app2.pdf]

Appendix 2. Questions on Sense of Control (Health and Retirement Study, 2012)

Perceived Constraints

Q. *Please say how much you agree or disagree with the following statements.*

Q22a I often feel helpless in dealing with the problems of life.

Q22b Other people determine most of what I can and cannot do.

Q22c What happens in my life is often beyond my control.

Q22d I have little control over the things that happen to me.

Q22e There is really no way I can solve the problems I have.

Response categories: 1=Strongly disagree, 2=Somewhat disagree, 3=Slightly disagree, 4=Slightly agree, 5=Somewhat agree, 6=Strongly agree

Perceived Mastery

Q. *Please say how much you agree or disagree with the following statements.*

Q23a I can do just about anything I really set my mind to.

Q23b When I really want to do something, I usually find a way to succeed at it.

Q23c Whether or not I am able to get what I want is in my own hands.

Q23d What happens to me in the future mostly depends on me.

Q23e I can do the things that I want to do.

Response categories: 1=Strongly disagree, 2=Somewhat disagree, 3=Slightly disagree, 4=Slightly agree, 5=Somewhat agree, 6=Strongly agree
